# Ranked prediction of p53 targets using hidden variable dynamic modeling

**DOI:** 10.1186/gb-2006-7-3-r25

**Published:** 2006-03-31

**Authors:** Martino Barenco, Daniela Tomescu, Daniel Brewer, Robin Callard, Jaroslav Stark, Michael Hubank

**Affiliations:** 1 Institute of Child Health, University College London, Guilford Street, London WC1N 1EH, UK; 2 CoMPLEX (Centre for Mathematics and Physics in the Life Sciences and Experimental Biology), University College London, Stephenson Way, London, NW1 2HE, UK; 3 Department of Mathematics, Imperial College London, London SW7 2AZ, UK

## Abstract

Full exploitation of microarray data requires hidden information that cannot be extracted using current analysis methodologies. We present a new approach, hidden variable dynamic modeling (HVDM), which derives the hidden profile of a transcription factor from time series microarray data, and generates a ranked list of predicted targets. We applied HVDM to the p53 network, validating predictions experimentally using small interfering RNA. HVDM can be applied in many systems biology contexts to predict regulation of gene activity quantitatively.

## Abstract 2

Hidden Variable Dynamic Modelling is a new approach to microarray analysis that quantitatively predicts the regulation of gene activity.

## Background

In order to understand how gene networks function, it is necessary to identify their components and to quantitatively describe how they relate to one another [1-3]. Subsequent prediction of gene network behavior requires identification of important parameters and variables, and estimation or measurement of their values during a response [4-6].

Experimental approaches can be applied to identify network components. For example, protein binding arrays and chromosome immunoprecipitation can be applied to identify transcription factor (TF)-binding sites and therefore infer TF targets [7-10]. However, these approaches give a static view of the system. Binding sites identified *in vitro *may not be available *in vivo*, and different regulators may be active in different cellular systems. Furthermore, purely experimental approaches cannot predict in a quantitative manner, and with statistical confidence, the dynamics of network activity without making an impractical number of experimental observations [11].

Insight into the dynamic relationships present in a transcriptional response can be gained by running time series of microarrays [3,11,12]. Currently, analysis of this type of datum chiefly relies on clustering or correlation methods. The assumption is that groups of genes with similar expression profiles over time are likely to be regulated by the same TF. Although clustering approaches have been applied with some success, they are limited and inaccurate. Genes with different profiles may still be regulated by the same TF, and many genes included in clusters may be regulated by other factors. Clustering approaches typically do not generate confidence statistics about the validity of individual predictions, and therefore they can neither rank candidates nor distinguish between true and false targets.

Importantly, because clustering is based on only the expression time profile, the influence of other important factors required to reconstruct gene network activity is not taken into account. For example, transcript degradation rates, the sensitivity of a gene to a TF (or affinity of binding to the promoter), and the activity of the TF itself all contribute to the overall transcriptional output. Where clustering methods alone are applied, these quantities remain hidden in the data and are likely to confound any attempted analysis. As a consequence, microarray experiments typically return a list of targets based on expression level alone, and prioritization of genes of interest depends chiefly on researcher intuition.

An alternative strategy is to use a mathematical model of the network dynamics to provide a framework for the analysis of the expression time profile. Several types of model have been applied at different levels of complexity ranging from parts lists to dynamic models [3,11,12]. In theory, modeling can be applied to reconstruct a gene network in a quantitative manner [3,11,13]. The advantage of such an approach is that all of the important mechanisms that affect transcript levels can be taken into account simultaneously. Statistical confidence intervals can then be calculated, which allow the prediction of transcriptional targets with a specified statistical significance. As a result it is possible to predict how network regulation would change in response to differing conditions, allowing the optimal targeting of expensive experimental approaches.

We therefore developed a mathematical approach that uses information from a dynamic microarray time series data set to estimate, with confidence intervals, key parameters and hidden variables, specifically TF activity profiles. We define TF activity in terms of the positive effect that the TF has on transcription of its targets. We chose as a model experimental system the transcriptional response to ionizing irradiation. Ionizing radiation induces DNA damage, which in turn activates the p53 response [14]. p53 is a transcription factor and tumor suppressor, but it is only one of several TFs activated by DNA damage [15,16].

Our analysis method allows quantitative prediction, with confidence, of transcripts that are upregulated by p53 in the complex response, without the need for very large numbers of experimental observations. We have made use of prior biologic information (known p53 targets) to construct a mathematical model of gene regulation, calculated confidence intervals using a highly efficient novel approach, and anchored the model by including a surprisingly small amount of additional biologic information. We show that the model outperforms a clustering approach in terms of accuracy of target prediction, and we successfully tested model predictions with a separate experimental data set.

## Results

### A model of transcription factor-dependent gene transcription

We grew and irradiated a human leukemia cell line (MOLT4) containing functional p53 and harvested protein and RNA at regular intervals after irradiation. The time course was performed in triplicate, and Affymetrix U133A microarrays (Affymetrix Inc., Santa Clara, CA, USA) were run to measure the global transcriptional response. Before irradiation, we assumed the p53 network to be in equilibrium (that is, that the rate of change in its constituents is zero). Irradiating the cells disrupts the equilibrium and activates transcription of numerous p53 target genes. The rate at which p53-dependent mRNA transcripts accumulate depends on the basal transcription rate of a target gene, the sensitivity of the gene to p53, the level of activity of p53, and the transcript degradation rate. We can connect these factors to represent the overall behavior of the system. The time evolution of each gene transcript is described by the following non-autonomous linear differential equation for the rate of change in transcript concentration x_j_(t) of gene j at time t:



Where B_j _is the constant basal transcription rate of j; S_j_f(t) is the transcription induced by p53, composed of a constant S_j_, which is the sensitivity of gene j to p53, and f(t), which is the activity of p53 at time t; and D_j_x_j_(t) is a degradation term, with D_j _being a constant degradation rate. For a full description of the model, see Mathematical methodology (below).

### Deriving the hidden activity profile of p53

In order to predict whether a gene is likely to be a p53 target, it is necessary to estimate its sensitivity (S_j_) to p53 and to ensure that parameter values can be found that, when combined in the model equation, result in an expression profile similar to the experimentally determined profile. However, the p53 activity f(t) is not experimentally available and is the key 'hidden variable' in the system. To estimate this profile we used prior biologic knowledge rather than adopting a 'black box' approach. We selected a small training set of five known p53 targets (*DDB2*, *p21*^*WAF*1/*CIP*1^, *SESN1*/*hPA26*, *BIK*, and *TNFRSF10b/TRAILreceptor 2*) [17-22] and used the microarray time series observations for this set to derive the p53 activity profile f(t), and the parameter values of basal transcription rate, sensitivity to p53, and degradation rate. These values and their confidence intervals were obtained by applying Markov Chain Monte Carlo (MCMC) with a Metropolis-Gibbs sampler [23] (see Mathematical methodology, below). Normally, the calculations involved in these estimations are very demanding on computer time. In terms of systems biology, in which many such calculations are likely to be linked, this poses a major barrier to network analysis. We therefore discretized the model equation and devised a fast matrix-based algorithm to solve it efficiently (see Mathematical methodology, below).

**Figure 1:**
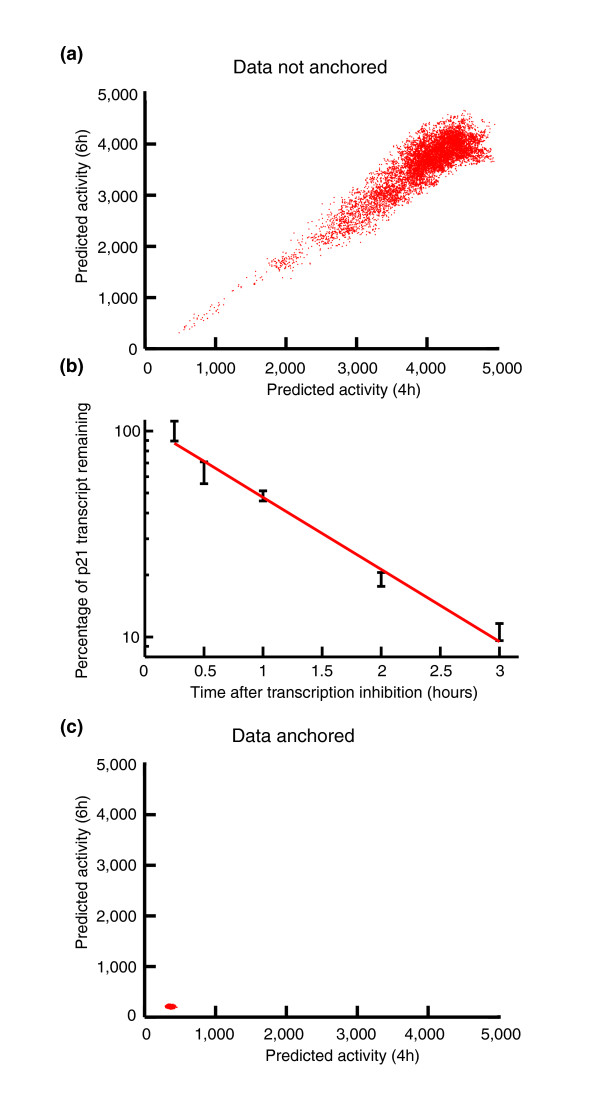
Model based estimation of activity profile of p53. **(a) **Markov Chain Monte Carlo output for potential transcription factor activity profile values for first time series replicate at 4 hours (*x *axis) and 6 hours (*y *axis). **(b) **Concentration of *p21*^*WAF*1 ^transcript determined by real-time polymerase chain reaction after addition of actinomycin D (10 μg/ml) to irradiated (5 Gy, 4 hours) MOLT4 cells cultured in RPMI. Expressed as percentage of initial concentration. **(c) **Using the degradation rate of *p21*^*WAF*1 ^dramatically restricted the range of solutions to the Markov Chain Monte Carlo.

Initial estimates of the parameters and the hidden profile f(t) exhibited a very high degree of variance. Repeated modeling of artificial data indicated that this was a general characteristic of the model and not peculiar to the particular experimental data set. We noticed that the estimates were highly correlated with each other (Figure 1a). This suggested that experimentally determining the value of one additional parameter might constrain the others and so reduce the overall variance. We therefore measured the rate of degradation of one transcript (*p21*^*WAF*1/*CIP*1^) using quantitative polymerase chain reaction (PCR; Figure 1b). We found that this single measurement was sufficient to reduce dramatically the variance and greatly improve the final estimates (Figure 1c). We term this process 'data anchoring'. We found that obtaining the degradation rate of any element in the training set was equally sufficient to anchor the model, provided that the same gene was also used as the reference point for estimating sensitivity (see Mathematical methodology, below). The inclusion of the degradation rates of more genes did not significantly improve parameter estimation.

**Figure 2:**
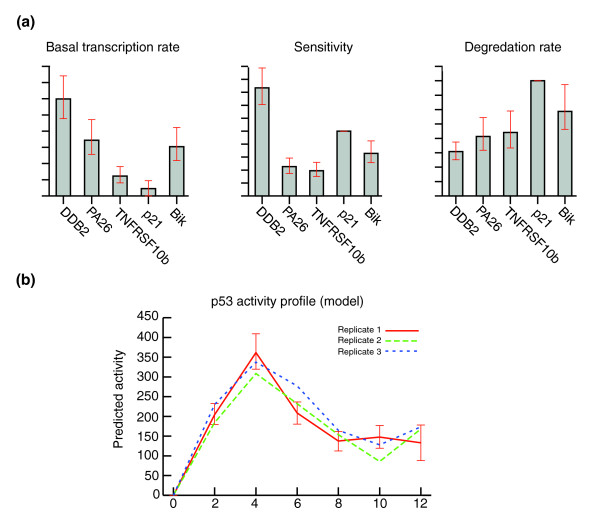
Parameter estimation for a training set of five known p53 targets. **(a) **The model equation was solved to estimate values for the parameters basal transcription B_j _sensitivity S_j_, and degradation D_j _for the five p53 targets *DDB2*, *p21*^*WAF*1/*CIP*1^, *SESN1*/*hPA26*, *BIK*, and *TNFRSF10b/TRAILreceptor 2*. **(b) **Simultaneously, the activity profile f(t) of p53 was derived from three separate microarray time courses.

**Figure 3:**
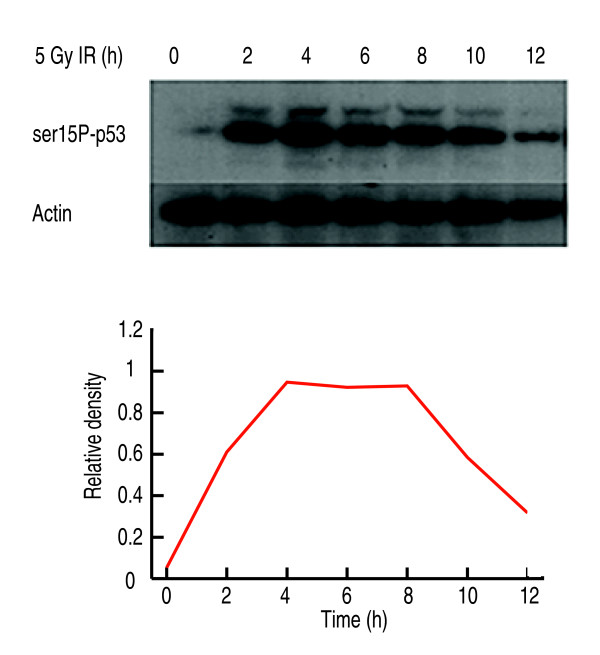
Experimentally determined p53 activity profile. The activity profile of p53 was measured by Western blot to determine the levels of ser-15 phosphorylated p53 (ser15P-p53). ser-15 phosphorylation is a measure of p53 activity. IR, ionizing radiation. IR, ionizing irradiation.

Incorporation of the degradation data allowed efficient estimation of the parameters B_j_, S_j _and D_j_, and the p53 activity profile f(t) for the training set of known targets (Figure 2). This process was performed simultaneously on three replicate time series to improve the robustness of the outcome (Figure 2b). We found that the model-estimated profile approximated the experimentally determined activity profile based on measuring p53 phosphorylation at serine 15 [24] (Figure 3). The profiles show a close match early in the response, but the model predicts a more rapid decline in activity. This discrepancy can be explained by the operation of other regulatory mechanisms that affect p53 activity but not concentration, for example relocation of phosphorylated p53 to the cytoplasm [25].

### Optimization of the model

**Figure 4:**
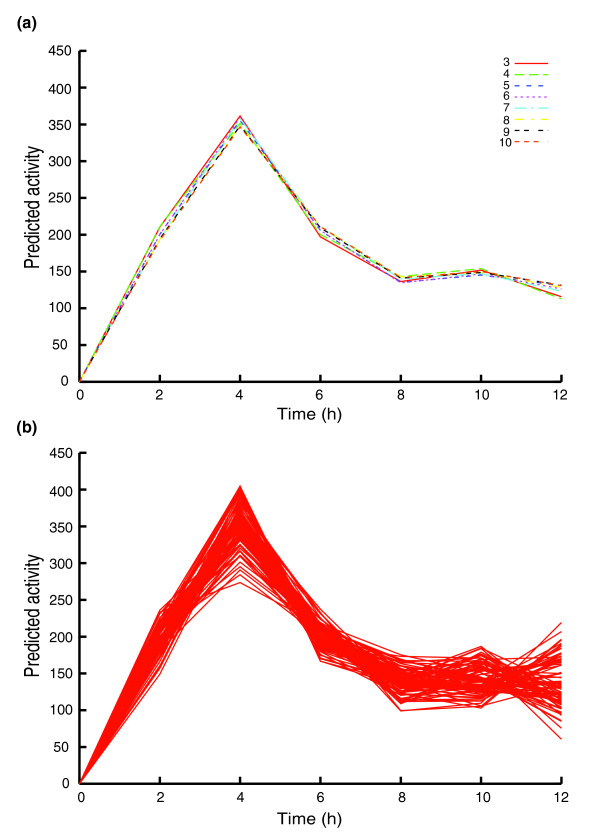
Choice and number of training set genes does not significantly affect the predicted activity profile. **(a) **Predicted activity profile of p53 derived using different numbers of known targets in the training set, from three to ten genes. **(b) **Predicted activity profile of p53 derived using 100 combinations of three randomly selected training set genes from a pool of 10 known targets.

The use of a training set of known targets takes advantage of the fact that prior biologic knowledge exists for many TFs. Because the p53 response is well studied, we were able to examine the optimum model requirements. We found that three training genes are sufficient for the model to make accurate parameter estimates (Figure 4a). The inclusion of more (up to ten) genes narrowed the confidence intervals but the improvement was small beyond five genes. We also found that inclusion of genes not regulated by p53 (for example *TNFSF10*) led to a poor gene-specific model score, enabling these genes to be excluded from the training set. We found the method to be very robust, and the exact choice of target genes does not appear to affect estimation greatly, providing that the measurement error is not excessive (namely, the detection *P *value should be below 0.001 for Affymetrix data) and that the anchoring gene is clearly differentially regulated (Figure 4b; also see Mathematical methodology, below).

### Prediction of p53 targets using hidden variable dynamic modeling

Once we had constructed the estimate for the key 'hidden variable', namely the p53 activity profile f(t), we were able to apply the model to the remaining expression data to predict p53 targets. Data was filtered to identify upregulated and detected genes (754 in total). These were then tested to determine how well they fitted the model of activation by p53. We derived a score M (> 0) based on the closeness of experimental data to model predictions (in which lower scores are better). Because nonchanging genes with a flat profile would also fit the model, another score was computed that captures the predicted sensitivity to p53. This sensitivity score is a measure of how significantly S_j _differs from zero, represented by a Z score. Z scores are the distance between the observed value and the population mean in units of standard deviation, and are therefore a measure of estimation robustness. Z scores are inversely related to *P *values (see Materials and methods, below).

**Figure 5:**
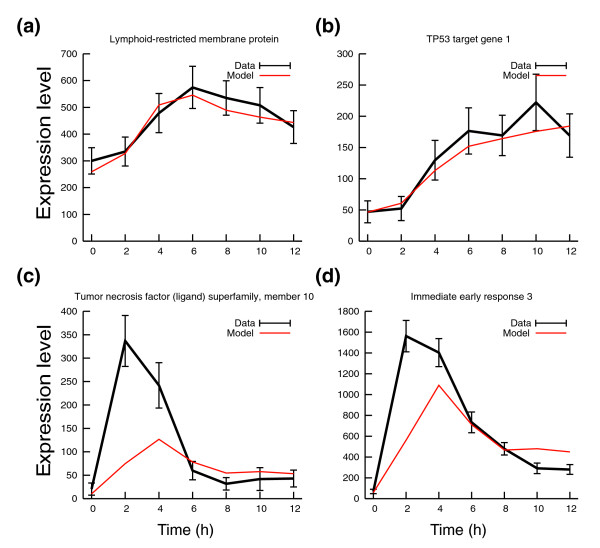
Hidden variable dynamic modeling screening of upregulated genes. Model predicted profile (red) and experimental expression profile (black) of typical genes representing two classes of model prediction (class 1 and class 2). **(a) **Class 1 genes with good model score (M < 100) and high sensitivity *P *value (sensitivity Z score > 2; for example *LRMP*). **(b) **Class 1 genes with atypical expression profiles (for example, *p53TG1*); this profile occurs because of a low predicted degradation rate. **(c,d) **Two class 2 genes with low model score (M > 100) but high sensitivity *P *value (sensitivity Z score > 2; for example, *TNFSF10 *and *IER3*).

We ranked the model scores, first in terms of model fit and then on predicted sensitivity to p53. Three broad classes of upregulated genes could be discerned, the composition of which depending on the stringency of the M score and sensitivity Z score threshold applied. At thresholds of M < 100 and sensitivity Z > 2 (and degradation estimates limited to 0.1/hour < D_j _< 5/hour), class 1 consisted of 237 genes that fitted the model well and exhibited high probability of p53 sensitivity, exemplified by *LRMP *and *p53TG1 *(Figure 5a,b). Class 1 genes were therefore most likely to include genes regulated by p53, with the probability of sensitivity being the key indicator. As expected, the five known targets composing the training set were found among the 20 highest scoring genes (ranked by decreasing sensitivity Z score), alongside other established p53 targets and genes not previously known to be p53 regulated (Table 1).

Under the same thresholds, in a second class of 105 genes a relatively high sensitivity score was achieved despite a poor model fit, as in the case of *TNFSF10 *(*TRAIL*) and *IER3 *(Figure 5c,d). The model attempts to accommodate genes strongly regulated by factors other than p53 by varying degradation and sensitivity scores, which often results in apparently high sensitivity predictions. However, the poor overall model fit suggests that class 2 genes are either completely independent of p53 or exhibit more complex co-regulation.

Genes in class 3 have either poor sensitivity or poor model score (*M *> 100, sensitivity *Z *< 2), or both. The majority of the 412 genes in this group are likely to be regulated independently from p53 in a manner that exhibits no similarity to the p53 activity profile. However, class 3 will also include genes that are p53 dependent but that are not distinguishable by the model.

### Verification of model predictions using small interfering RNA to p53

**Figure 6:**
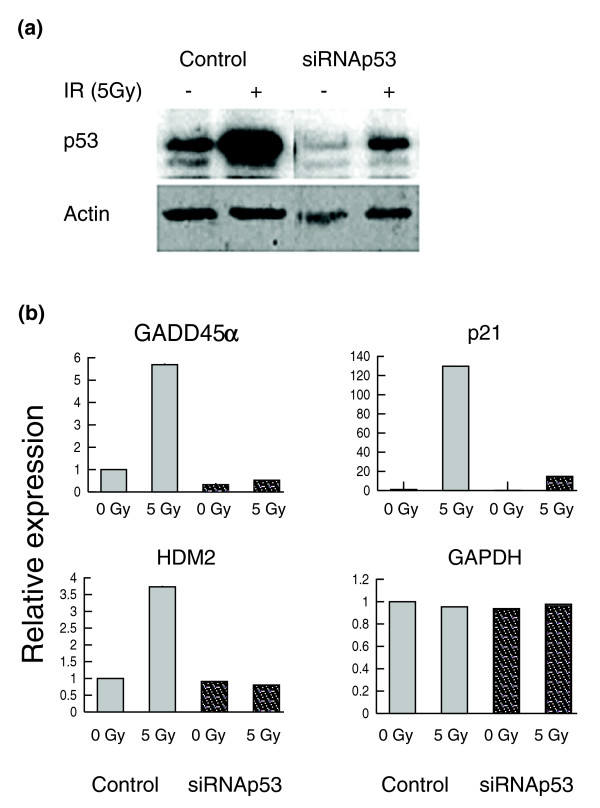
Small interfering (si)RNAp53 reduces p53 protein levels and transcription of p53 target genes. **(a) **Transfection of siRNAp53 reduces p53 protein levels below control values. **(b) **Real-time quantitative polymerase chain reaction measurement of three p53 target genes (*GADD45α, p21*, and *HDM2*) and a control gene (*GAPDH*) after transfection of siRNAp53 and irradiation. IR, ionizing irradiation.

To validate the predictions made by the model, we transfected MOLT4 cells with small interfering (si)RNA to p53 to deplete p53 protein to below control levels (Figure 6a) [26]. siRNAp53 substantially reduced ionizing irradiation-induced increases in the transcripts of three p53 target genes, namely *HDM2*, *P21*, and *GADD45α *(Figure 6b). We then ran microarrays to measure the effect of siRNAp53 on the transcriptional response to irradiation at the whole genome level. Validation was carried out at 4 hours to maximize the number of p53 targets and to minimize the inclusion of secondary targets. Data were filtered to identify those genes that were upregulated in both the time course and in the pSuper transfected control at 4 hours (see Materials and methods, below). This identified a total of 162 genes that were upregulated significantly by irradiation at 4 hours.

**Figure 7:**
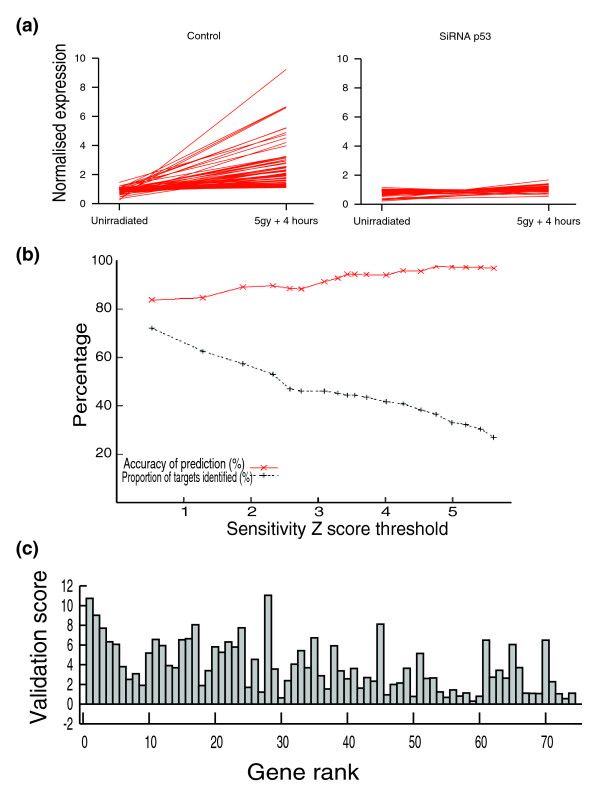
Model validation. **(a) **Effect of small interfering (si)RNAp53 on irradiation (5 Gy) induced change in transcript levels at 4 hours of the 74 class 1 genes. **(b) **Effect of altering S_j _Z score threshold for class 1 on proportion of true targets identified (% of p53 upregulated genes at 4 hours predicted; black line) and accuracy of class 1 predictions (percentage of predictions made that were verified by siRNAp53; red line). Accuracy and proportion of the data explained reveal an inverse relationship. **(c) **Individual comparison of the effect of siRNAp53 on 74 class 1 genes with the best M and p53 sensitivity S_j _score, ranked by sensitivity. Bars represent the validation score, a Z score measuring the effectiveness of siRNAp53 on reducing post-irradiation upregulation of transcript. Higher scores indicate effective blocking of the response.

To quantify sensitivity to siRNAp53 at the individual gene level, we computed new Z scores that measured the difference between genes upregulated by irradiation in control cells and those upregulated in siRNAp53 treated cells. For clarity, these are referred to as validation scores. The higher the validation score, the more effectively siRNAp53 eliminates change in transcript concentration, and so the more likely the gene is to be dependent on p53. Seventy-four of the 162 4-hour-upregulated genes were predicted by the model to be p53 targets because they fell into class 1 (M < 100 and sensitivity Z score > 2). Of these 74, 66 (90%) exhibited high (Z > 1) validation scores (namely sensitivity to siRNAp53), confirming that they are p53 targets (Figure 7a). This figure rises to 73 out of 74 (98%) if a lower sensitivity Z score threshold (> 0.5) is applied or falls to 39 out of 74 (53%) if the sensitivity Z score threshold is set at 3. Higher sensitivity Z score thresholds therefore result in greater accuracy but at the expense of identifying a lower proportion of the targets (Figure 7b). Sensitivity Z score correlated well with validation score, indicating that predicted rank of p53 targets reflected the strength of p53 regulation (Figure 7c).

Thirty upregulated (4 hours) genes fell into class 2 (M > 100 and sensitivity Z score > 2). As expected, the response of class 2 genes to siRNAp53 was divided. Fourteen genes, including *TNFSF10 *(*TRAIL*), remained unaffected by siRNAp53, showing them to be p53 independent/irradiation dependent. Sixteen class 2 genes were affected to some degree by the treatment, confirming predictions that this group included co-activated or co-repressed genes such as *IER3*, which is known to be synergistically regulated by nuclear factor-κB and p53 [27]. The remaining 58 upregulated (4 hours) genes fell into class 3, 34 of which were affected by siRNAp53.

Overall the Z score for S_j _(sensitivity to p53) was a good discriminator for identifying p53 targets. The model was able to predict with confidence, and at high accuracy, 66 out of 115 (57%) genes verified as p53 targets at 4 hours, based on a sensitivity Z score threshold of 2. A further 16 class 2 genes exhibited evidence of co-regulation, suggesting an explanation for 71% of the interpretable data. Many of the remaining class 3 targets were expressed at low levels, or exhibited low (> 1.5-fold) levels of differential expression. This raises questions about their biologic significance, and suggests that the true success rate of hidden variable dynamic modeling (HVDM) is actually higher than reported above. A larger number of replicates would be required to be confident of the status of class 3 genes.

**Figure 8:**
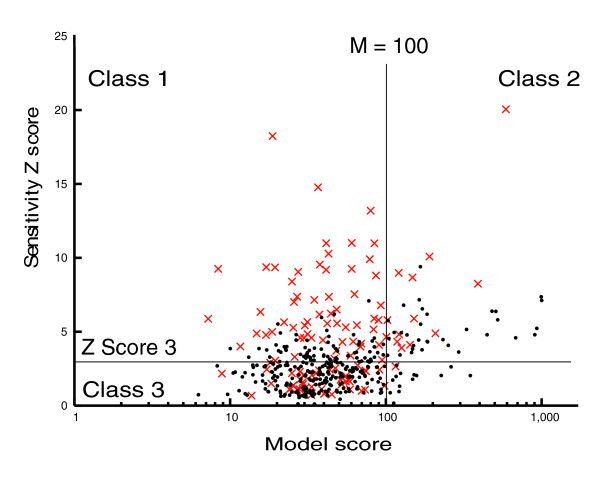
Model performance. Distribution of 459 upregulated genes that pass degradation filter based on model score and predicted sensitivity to p53. S_j _Z score = 3 and model = 100 thresholds are shown. A total of 115 Genes verified as p53 targets at 4 hours are shown in red.

As seen for the validation data set, tightening thresholds (by choosing a higher sensitivity *Z *score) results in more confidence that the targets are regulated by p53 but at the cost of explaining a lower percentage of the data (Figure 7). When applied to the entire upregulated data set, HVDM can accurately predict a large number of p53 targets from a short time course without any further experimental input (Figure 8). These predictions included a number of genes not previously known to be p53 targets, including *CD38*, DENN-domain protein *FLJ22457*, *CROT*, *GLS2*, *HERC5*, *ASCC3*, *LRMP*, and *CIKS*/*ACT1*/*TRAF3IP2 *(Table 1). siRNA validation at an early time point (4 hours) indicates that these genes are most likely to be direct targets. CD38 is best known as a prognostic marker in the leukemia B cell lymphocytic leukemia (B-CLL associated) with poor outcome. It functions as a powerful regulator of calcium dependent signaling via the generation of cyclic ADP ribose and NAADP+ (nicotinic acid adenine dinucleotide phosphate) Its regulation by p53 suggests a possible role for calcium-dependent signaling in the DNA damage response.

### Hidden variable dynamic modeling predicts p53 targets more accurately than does K means clustering

**Figure 9:**
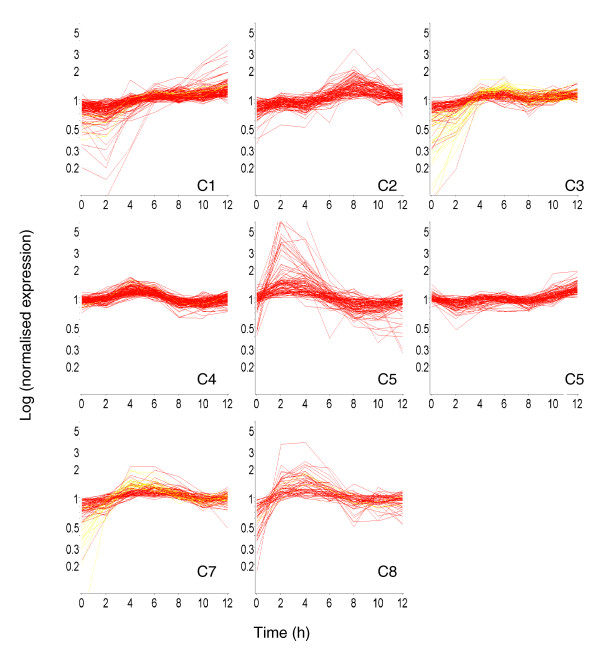
K means clustering of upregulated genes based on expression values. A total of 754 upregulated genes were optimally grouped into eight K means clusters (C1 to C8). The 50 best hidden variable dynamic modeling predictions (Table 1) are split among six clusters (highlighted in yellow). Accurate prediction of p53 targets is therefore not possible using K means at this level.

Since both HVDM and clustering approaches aim to identify TF targets, we compared our results with a typical clustering approach, namely K means clustering. From the 754 genes identified as upregulated by irradiation, HVDM generated a ranked list of predicted p53 targets based on model score and best sensitivity Z scores (Table 1). Forty-eight of the 50 highest ranked targets (96%) predicted by HVDM were confirmed by siRNA to be p53 targets. These 50 HVDM predicted target genes were divided by K means clustering between six of eight clusters, each with a distinct response profile (Figure 9). For example, the HVDM predicted target *TP53TG1 *has a late expression profile (cluster 1, Figure 9), along with seven other top 50 targets. This profile is quite different from the activation profile of p53 or its 'typical' correlated targets (Figure 5b). Only two genes were probably false positives.

K means clustering of the 754 detected and upregulated genes based on expression levels alone grouped the genes into eight clusters based on transcript time profile (Figure 9). Visual examination of the profiles suggested that one of these classes (cluster 7, Figure 9) was most similar to the p53 activity profile determined by Western blot (Figure 5b), and indeed this cluster contained many of the well known p53 targets (including *GADD45α*, *p21*, and *DDB2*). However, because clustering approaches typically do not provide confidence intervals, it is impossible to identify which genes within the cluster are most or least likely to be real targets. We found that 25 out of 79 genes in cluster 7 were verified as p53 targets in the siRNA experiment (32%; data not shown). Verified genes also occurred in cluster 1 (11 out of 135 (8%)), cluster 3 (35 out of 102 (34%)), cluster 4 (21 out of 120 (17.5%)), cluster 5 (3 out of 90 (3.3%)), and cluster 6 (20 out of 51 (39%)).

In summary, HVDM can generate an accurate list of p53 targets with different expression profiles, ranked by probability of sensitivity to p53. In contrast, although K means clustering generates clusters enriched or depleted in p53 targets, it fails to identify targets with different profiles, is unable to quantify the level of sensitivity of a gene to p53, and cannot distinguish between true and false p53 targets. We also assessed the performance of self-organizing maps (SOM) clustering, with a similar outcome. This is expected, given that all processes that cluster on expression profile alone are bound to suffer similar deficiencies. Predictions made by HVDM are therefore accurate, explain a significant proportion of true targets, give indications about potential for co-regulation, and provide an excellent basis for prioritization of downstream bioinformatics and experimental analysis.

## Discussion

We present here an approach based on a simple differential equation model that uses hidden information to partially reconstruct, with confidence intervals, the p53 target network. Our algorithm, which we term hidden variable dynamic modeling, operates on two levels. First, it offers a quantitative description of a TF output network at the genomic level. Second, it provides a practical resource to enable the prediction of targets and a probability based prioritization of array data for downstream analysis.

Mathematical modeling of gene networks has taken a variety of approaches [3,11,12]. At the genome level, topographic network reconstruction has been achieved using a variety of methods and data sources, including microarray data [1,28-30]. In contrast, dynamic modeling has typically been limited to short pathways or feedback loops because of the complexity associated with estimating high dimensional models [11]. Some attempts to group network behavior into modules for dynamic modeling have been successful [13]. Others have attempted dynamic modeling of whole microarray data sets using differential equation models to derive transcriptional profiles [5,6,31]. However, these interesting studies stop short of calculating confidence intervals that take into account measurement error and variability [31,32]. Without these measurements, the reliability of the model cannot be assessed. Neither do they test predictions made by the model by experimentation.

Most microarray data are analyzed by subjecting them to various levels of statistical filtering to identify differences between two or more conditions. The resultant list of genes may then be segregated according to gene ontology using various tools designed for this purpose [33]. It is assumed that co-regulation of genes with a particular ontology is of interest, but this may be misleading and certainly cannot predict targets of a particular TF. Correlation approaches that cluster genes that exhibit a similar time course expression profile are more successful [34], but they are often inaccurate and miss many genuine targets with a different profile. The advantage of our approach is that it can predict genes with any profile as targets of the same TF.

Complex data sets contain hidden information about gene regulatory networks [35]. It has also been suggested that the use of prior biologic knowledge can improve the reconstruction of genetic networks [36]. In generating our model, we used a small amount of knowledge about TF targets to derive the activity profile of p53 and then applied this to partially reconstruct the p53 target network. Our model makes the assumption that, given a short time course, much of the network behavior can be explained using linear modeling, and our verification experiment strongly supports this assertion (Table 1). However, it is likely that some genes respond to p53 in a nonlinear manner, for instance as a result of saturation and/or threshold effects. Future extensions of our model to include these terms may explain an even higher proportion of the behavior (work in progress). It should also be noted that the model would be unable to distinguish between TFs with identical activity profiles. Combination of HVDM with experimental approaches or other *in silico *methods such as the identification of TF-binding sites could help to resolve this issue [37-39]. The current model is only able to account for direct effects of the controlling TF, which is reasonable for the short time course employed in our studies. Future modifications to the model will permit modeling of secondary effects, namely genes upregulated at late time points that may be targets of targets.

HVDM correctly predicted the majority of p53 targets, including all of the well known examples, directly from time series measurements of a complex response. HVDM was also able to identify, with associated probability, genes that had not previously been identified as p53 targets. Several previous studies have aimed to identify p53 target genes on a genome wide level using microarrays. Zhao and coworkers [22] identified p53 targets by using a Zn^2+^-inducible p53 construct containing a metallothionein promoter. In this case, the specific induction of p53 required the establishment of a complex and artificial *in vivo *system. p53 targets could not be directly extracted from ionizing irradiation or ultraviolet irradiation experiments alone. Also, targets induced in the artificial system differed significantly from those induced by ionizing irradiation or ultraviolet, indicating that simple artificial systems cannot replicate the behavior of complex activities during a physiologic response. In another approach, Kannan and coworkers [40] employed a temperature sensitive p53 to identify p53 dependent transcription and used cycloheximide to distinguish between primary and secondary targets. However, again, a complex artificial system was required. Furthermore, temperature and cycloheximide are both likely to affect the resultant transcription patterns, and because the data cannot be ranked the reliability of many targets would require additional experimental verification. HVDM has the advantage that ranked probability based target lists can be extracted from complex data without having to isolate each factor experimentally.

We observed that genes that were affected by siRNAp53 but not predicted by the model typically exhibited expression levels close to the detection threshold or low levels of differential regulation, or were poorly hybridizing alternative probe sets for genes already predicted by the model to be targets. The biologic significance of many apparent targets not identified by the model is therefore questionable. The ability to provide ranked lists of predicted (class 1) targets with a high degree of confidence, and based on the minimum of input data, will allow researchers to make optimal use of their resources. Such prioritization has been lacking in microarray data analysis and has hampered the efficient interpretation of array experiments.

It is important to note that the model is dynamic. It not only identifies targets but also predicts network behavior in response to changing conditions or altered parameters. For example, treatment with a drug that alters p53 activity could potentially be modeled entirely *in silico *based on its effects on expression of the training set of target genes. This may have implications for predicting the consequence of clinical or experimental treatments [41].

## Conclusion

We addressed the problem of extracting hidden information from time series microarray data. We present a method that models the p53 target network following DNA damage, in which we use prior biologic information (a training set) to construct a mathematical model of the transcriptional response to DNA damage in MOLT4 cells. We have also developed a method to calculate confidence intervals for parameter estimates in a highly efficient manner. We found that the inclusion of a surprisingly small amount of additional biologic information was necessary to anchor the model. Most importantly, we then successfully tested the model predictions with an entirely separate experimental data set.

Our model accurately predicted a significant proportion of transcriptional targets of p53 and explained their behavior. The model identified genes not previously known to be p53 regulated, and it is more widely applicable and more accurate than correlation or clustering methods because it considers degradation rates as well as transcript accumulation profiles. Furthermore, HVDM can extract hidden information from small data sets in which experimental methods would require an impractical number of observations. Finally, HVDM allows the probability-based prioritization of microarray data, permitting researchers to exclude irrelevant information and rapidly focus on the networks of interest.

HVDM can be applied to any large time series data set in which identification of hidden variables can reveal critical information about network dynamics. The approach is quantitative and predictive, and demonstrates that combining mathematical modeling with experimental observations can help to unravel complex relationships in biologic systems.

## Materials and methods

### Biological methods

#### Cell lines and reagents

Human MOLT4 cells (T cell acute lymphoblastic leukaemia) were obtained from the National Institute for Biological Standards and Controls (Potters Bar, Herts, K; CFARP011) and cultured in RPMI, 10% fetal calf serum and L-glutamine, plus antibiotics. p53 genotype was determined by sequencing to verify wild-type status. p53 accumulation was monitored after irradiation by quantitative Western blotting, and regulation of known p53 targets (*p21*, *GADD45α*, and *MDM2*) following p53 activation by ionizing radiation was established to confirm p53 wild-type behavior (data not shown). Western blots were probed against total p53 (Santa Cruz Biotechnology Inc. Santa Cruz, CA, USA), phospho-p53 (Cell Signalling Technologies, Danvers, MA, USA), and actin (Santa Cruz). Proteins were detected using enhanced chemiluminescence (ECL+; GE Healthcare, Chalfont St Giles, Bucks, UK) and quantified by densitometry.

#### Microarray time course

Cells in log phase (1 × 10^6^/ml) were γ-irradiated with 5 Gy at room temperature at a dose rate of 2.45 Gy/minute with a ^137^Cs γ-irradiator. Cells were harvested at 0, 2, 4, 6, 8, 10 and 12 hours, and RNA and protein were extracted (Trizol; Invitrogen, Paisley, UK). RNA and cRNA quantity and quality were determined by Nanodrop spectophotometer and Bioanalyser 2100 (Agilent, Wokingham, Berks, UK). Affymetrix U133A arrays (Affymetrix, Sanat Clara, CA, USA) were hybridized as standard. Array quality was determined using R and GCOS .rpt file values. The time course was replicated three times from independent cell preparations.

#### Microarray data analysis

Microarray data were summarized using the MAS5.0 algorithm (Affymetrix). Signal distribution was assessed using Genespring 6.1 (Agilent), and data were normalised to the median and log transformed for further analysis. For modeling applications, rescaled MAS5.0 data were analyzed using C code [42] (see Mathematical methodology, below). Data are available in MAGE-ML format via ArrayExpress (European Bioinformatics Institute) or on request.

#### Prediction of p53 targets

Data were filtered to identify 754 genes that were confidently upregulated by ionizing radiation (but not necessarily by p53) in at least one time point, and to exclude control genes (for example, spike ins). We excluded genes predicted to have a biologically impossible degradation rate (either close to zero (< 0.01/hour) or with too short a half-life (rate > 5/hour)). Next, we calculated the sum M of weighted differences between the model predicted profile and the experimentally determined transcript profile. Finally, the confidence that the transcript was sensitive to p53 activation was assessed by determining the probability that each individual sensitivity S_j _was equal to 0. The modeling and statistical techniques used to compute these indicators are described extensively below.

#### Real-time quantitative polymerase chain reaction

MOLT4 cells were irradiated with 5 Gy and incubated at 37°C for various time periods. First strand cDNA was prepared (Invitrogen) and used as a template in PCR reactions with predeveloped target assays (Applied Biosystems, Foster City, CA, USA): *p21, HDM2, GADD45*, and *GAPDH*. Target and reference were amplified in separate wells in a 96-well setup with three replicates for each reaction on ABI Prism SDS 7000 (Applied Biosystems), using default cycling conditions. Change in gene expression was calculated using 2^-dCT^, where dC_T _is the mean of C_T _(threshold cycle number) values obtained from the triplicate samples at each time point.

#### Small interfering RNA experiments

Cells were transfected with siRNAp53 (DNAEngine, Oligoengine, Seattle, WA, USA) or the vector-only control (pSuper, Oligoengine), together with a marker plasmid (pcDNAneo-GFP) using electroporation. GFP-positive cells (40-50%) were sorted 24 hours after transfection on a Mo-Flo FACS sorter (Cytomation, Fort Collins, CO, USA) to a purity in excess of 98%. Forty-eight hours later sorted cells were irradiated with 5 Gy or mock irradiated and incubated for 4 hours at 37°C. RNA and protein were then prepared and processed for real-time quantitiative PCR, protein analysis, and microarray. For verification, data was filtered to include genes detected (Affymetrix *P *< 0.04 at t = 4 hours) and changed (Z score > 1) at 4 hours in both the time course and in the pSuper control, and to remove genes whose siRNAp53 call was caused mainly by differences in basal transcription levels (removing 28 out of 190 genes).

### Mathematical methods

#### Model formulation

We assume that the transcript concentration x_j_(t) of gene j satisfies the following time-dependent linear differential equation:



This assumes that the transcript is degraded proportionally to its concentration, with the degradation rate D_j_. The production term B_j _+ S_j_f(t) comprises a basal transcription rate B_j_, which may be increased proportionally by the TF activity f(t). S_j _is the sensitivity of gene j to that TF. Our overall aim is to estimate the parameters B_j_, S_j_, and D_j _from the microarray data [_j_(t_i_)], and in particular the sensitivity S_j_. If S_j _is not significantly greater than zero, then gene j is not regulated by the TF, whereas if S_j _is large and significantly different from zero then the TF has a very strong effect on that gene.

The activity level f(t) of the TF (p53) is unknown. In order to estimate the hidden variable f(t), we need to parametrize the function f and estimate the resulting parameters. This raises the problem that if we try to estimate the unknown parameters in Equation 1 for single gene j, then we have more unknown parameters than we have data points. The unknowns are the three parameters B_j_, S_j_, D_j_, and the n + 1 values f(t_0_) ... f(t_n_), as compared to the n + 1 observed data points _j_(t_0_) ... _j_(t_n_). (The term _j _is the experimental measurements of gene j, composed of x_j _+ ε, where ε is the measurement error.)

We observed that the equations for different genes are coupled by the level f(t) of the TF. Thus if we measure m genes simultaneously with microarrays, then we have m(n + 1) measurements _j_(t_i_) for j = 1 ... m and i = 0 ... *n*, but there are only 3 m + n + 1 unknowns B_j_, S_j_, D_j _for j = 1 ... m and f(t_0_) ... f(t_n_). If the number of time points n is sufficiently large, then m(n + 1) = 3 m + n + 1, and we are able to estimate the unknowns using standard optimization methods. In practice we applied the model to replicate measurements that requires a modification to this approach (see Additional data file 1).

As the model stands, different parameter combinations could give rise to identical solutions for x_j_(t). This is because both the origin and the scaling of the unobserved TF activity are arbitrary. Suppose that f(t) = α(*t*) + β for some constants α ≠ 0 and β. Then Equation 1 becomes the following:



Where _j _= B_j _+ S_j_β and  = αS_j_. Because the TF is not observed, we have no way to distinguish between the models in Equation 1 and Equation 2. To overcome this ambiguity, we first set S_j _= 1 for one gene, in our case p21. This fixes α and removes the ambiguity from the remaining S_j _and reduces by one the total number of parameters to be estimated. Second, we assume that at the start of the experiment the activity level of the TF is 0. In other words, we set f(t_0_) = 0, which is sufficient to fix β and hence remove the ambiguity from the B_j_. It further reduces the parameter count by one.

Setting f(t_0_) = 0 has the effect of defining the basal transcription rate B_j _for each gene as the rate at the start of the experiment. We assume that the p53 network is in equilibrium before irradiation, and hence B_j _can be thought of as the equilibrium transcription rate. Similarly, f can be interpreted as the deviation from equilibrium of the transcription factor activity.

#### Discretizing the model

In a systems biology context it is necessary to screen thousands of potential targets. We therefore developed a very rapid numerical method for estimating parameters in Equation 1.

Since Equation 1 is linear, it is possible to obtain an analytic solution:



We found that parameter estimation by direct application of standard numerical schemes for the evaluation of integrals was too slow. These approaches also suffer from the requirement to define an appropriate functional form and parametrization of f(t).

Instead, motivated by collocation based approaches to boundary value problems for nonlinear differential equations, we chose to discretize the model directly, converting the problem into an algebraic one. We illustrate this approach using the simplest possible discretization scheme.

To estimate the parameters in Equation 1 we must evaluate the derivative, which we denote Δ_i_, on the left hand side at a specific time point t_i_. Knowing the values x_j_(t_i-1_), x_j_(t_i_), and x_j_(t_i+1_) at neighboring points, we computed the slope of x_j_(t) between t_i-1 _and t_i_, and the slope between t_i _and t_i+1_. We then combined these two values using an appropriate weighting and obtained an estimate for Δ_i _(for notational convenience we define x_i _= x_j_(t_i-1_)). If the time intervals are regular, so that t_i _- t_i-1 _= t_i+1 _- t_i_, then:



This is equivalent to fitting a quadratic polynomial to the three points, and then evaluating its derivative at t_i_. Higher order approximations can be obtained by using more points and fitting an appropriate polynomial. A suitable way of doing this is Lagrange interpolation [42]. This gives an explicit formula for a degree q - 1 polynomial P(t) passing through the q points (t_p_, x_p_) ... (t_i_, x_i_) ... (t_r_, x_r_), where r = p + q - 1:



Where



We call such an approximation a q-point approximation (so that the example above gives a three-point approximation). An approximation of the required derivative Δ_i _can now be obtained by differentiating P(t) at t_i_.



The right hand side is a linear function of x_0 _... x_n_. We shall denote the coefficients of this by A_ik_, so that:



Where we set A_ik _= 0 for k < p or k > r. (For a detailed calculation of A_ik_, see Additional data file 1.) We can then collate these various coefficients into a (n + 1) × (n + 1) matrix A. If we define the (n + 1) vectors as follows:

x_j _= [x_j_(t_0_) ... x_j_(t_n_)] = (x_0 _... x_n_)

f = [f(t_0_) ... f(t_n_)] = (f_0 _... f_n_)

1 = (1 ... 1)

Then our approximation of Equation 1 can then be written as follows:

Ax_j _= B_j_1 + S_j_f - D_j_x_j _    (6)

The formal solution is given by

x_j _= (A + D_j_I)^-1^(B_j_1 + S_j_f)

Where I is the (n + 1) × (n + 1) identity matrix. In practice we solve Equation 6 using the Loewr-Upper decomposition [42] of (A + D_j_I).

Our approach to the solution of Equation 1 is several orders of magnitude faster than a naïve approach to solving the differential equation using an explicit fourth order Runge-Kutta, with the typical step size that would be employed in such a case. It also has the advantage that it is not necessary to specify a functional form for f(t). In our case f(t) is simply represented by the discrete set of values (f_0 _... f_n_), that is by the vector f.

Although our approach implicitly integrates the differential equation using large step sizes (effectively t_i+1 _- t_i_), the unavoidable errors associated with estimating f(t) swamp any advantage gained by using smaller steps, and consequently there is no loss of accuracy in replacing Equation 1 by Equation 6. We validated this conclusion by generating artificial data and adding Gaussian noise of similar amplitude to that generally seen in microarray data. We found that the error in the parameter estimation induced by this noise overshadows the discretization error.

#### Model fitting

We employed the discretized model described in Equation 6 in two different ways. First, to estimate the TF profile f(t), we fit a microarray time series to a training set of five genes known to depend on p53 (*DDB2*, *p21*^*WAF*1/*CIP*1^, *SESN1*/*hPA26*, *BIK*, and *TNFRSF10b/TRAILreceptor 2*). In order to do this, we also needed to estimate the parameters B_j_, S_j_, and D_j _for these genes, although the estimated values are of no direct interest. We call the estimated profile obtained from this phase  = (_0 _... _n_). We then used the estimated profile  and applied the model to the transcription time series [_j_(t_0_) ... _j_(t_n_)] for each gene j in our data set to estimate B_j_, S_j_, and D_j_.

In each phase we employed a standard approach to fitting the unknown parameters. We define a candidate parameter vector, which in the first phase is given by the following equation:

μ = (B_1 _... B_m_, S_1 _... S_m_, D_1 _... D_m_, f_0 _... f_n_)

In the second phase it is given by:

μ_j _= (B_j_, S_j_, D_j_)

Equation 6 was solved for this set of parameters using the LU decomposition of (A + D_j_I). In the first phase f = (f_0 _... f_n_), with the f_i _taken from the candidate parameter vector μ. In the second phase we used  = (_1 _... _m_) obtained from the first phase. We then computed the error M_j _(depending on μ or μ_j_, respectively) for each gene between the model solution and the actual data:



We assume the measurement errors to be independent and normally distributed with standard deviation σ_j_(t_i_) for the observation at time t_i _of gene j. The calculation of σ_j_(t_i_) is detailed in Additional data file 1.

In the first phase the error over the training set (containing m genes) is then



To fit the model, we then varied μ or μ_j _in a systematic way to find the parameters that make M or M_j _as small as possible using a MCMC method [23]. This has the added advantage of also providing confidence intervals for the resulting parameter estimates. We assume that the measurement errors are Gaussian, giving a likelihood function proportional to exp (-M/2) or exp(-M_j_/2). A Metropolis-Gibbs sampler was then applied with this likelihood. Because neither B_j _nor D_j _can take negative values, the MCMC sampling was carried out in logarithmic space for these two parameters [(og(B_j_) and log(D_j_), respectively).

To improve the convergence speed of the MCMC scheme it is advantageous to make jumps in the parameter space that are commensurate with the different parameter scales. This is particularly the case in the first phase, in which the dimensionality of the parameter space is high. We estimated such scales by running partial MCMC schemes on each group of parameters in turn, before running the full MCMC scheme. Specifically, parameters were grouped into four sets: degradation, transcription, basal rates, and TF profile. For each set a scale was determined to achieve an acceptance rate of approximately 25%. A final tuning run was carried out on the whole parameter set in order to achieve the prescribed acceptance rate of 25%. The main MCMC was seeded with the minimum of M found from a standard optimization procedure (the Nelder-Mead simplex algorithm). A burn in of 10,000 iterations was applied before proper sampling. The final sample of 10,000 observations was extracted at random from the 10^7 ^iterations following burn in. We verified that these choices yielded good convergence.

In the second phase, in which the TF profile is known and there are only three parameters to determine, a long iteration run is unnecessary. We found that 10^5 ^iterations were sufficient to produce good convergence. Once again 10,000 observations randomly sampled from those iterations were used to compute the relevant statistics.

## Additional data files

The following additional data are included with the online version of this article: A Word document giving details of the rescaling of array data, derivation of the coefficients of the differential operator, extension of model fitting to replicate measurements, and estimation of the measurement error (Additional data file 1).

## Supplementary Material

Additional data file 1 A Word document giving details of the rescaling of array data, derivation of the coefficients of the differential operator, extension of model fitting to replicate measurements, and estimation of the measurement error Click here for file

## Figures and Tables

**Table 1:** Top 50 genes predicted by hidden variable dynamic modeling to be p53 regulated, ranked by sensitivity Z score

Gene title	Gene symbol	Affymetrix identifier	Model score (M)	Sensitivity (Z score)	RNAi validation score
**Damage-specific DNA binding protein 2, 48 kDa**	*DDB2*	203409_at	18.74	18.24	10.74
CD38 antigen (p45)	*CD38*	205692_s_at	36.69	14.77	9.02
**Ferredoxin reductase**	*FDXR*	207813_s_at	79.82	13.19	7.72
Hypothetical protein FLJ22457	*FLJ22457*	221081_s_at	60.45	11.01	6.33
**Tripartite motif-containing 22**	*TRIM22*	213293_s_at	41.36	10.99	6.07
Carnitine O-octanoyltransferase	*CROT*	204573_at	84.40	10.98	3.80
Glutaminase 2 (liver, mitochondrial)	*GLS2*	205531_s_at	42.83	10.28	2.52
**Leucine-rich repeats and death domain containing**	*LRDD*	219019_at	78.80	9.90	3.09
Hect domain and RLD 5	*HERC5*	219863_at	37.65	9.55	1.91
**Cyclin G**_1_	*CCNG1*	208796_s_at	17.04	9.37	5.18
**BCL2-interacting killer**	*BIK*	205780_at	19.43	9.35	6.57
Activating signal cointegrator 1 complex subunit 3	*ASCC3*	212815_at	60.34	9.26	5.93
**Sestrin 1**	*SESN1*	218346_s_at	8.37	9.25	3.90
**p53 target zinc finger protein**	*WIG1*	219628_at	41.33	9.19	3.70
**Tumor necrosis factor receptor superfamily, member 10b**	*TNFRSF10B*	209295_at	27.34	9.05	6.52
Chromosome 6 open reading frame 4	*C6orf4*	215411_s_at	86.45	8.81	6.64
**Cyclin-dependent kinase inhibitor 1A(p21)**	*CDKN1A*	202284_s_at	24.98	8.40	8.07
**Etoposide induced 2.4 mRNA**	*EI24/PIG8*	216396_s_at	88.04	8.20	4.09
*Mitogen-activated protein kinase kinase kinase kinase 4*	*MAP4K4*	206571_s_at	62.88	7.54	1.88
Lymphoid-restricted membrane protein	*LRMP*	204674_at	26.92	7.36	3.40
**Xeroderma pigmentosum, group C**	*XPC*	209375_at	43.09	7.36	5.80
TNF (ligand) superfamily, member 4 (Ox40L)	*TNFSF4*	207426_s_at	34.73	7.15	5.26
**Human cleavage/polyadenylation specificity factor**	*CPSF1*	33132_at	77.75	7.09	-1.44
AMP-activated protein kinase, beta 1 subunit	*PRKAB1*	201834_at	25.72	7.01	6.30
Transducer of ERBB2, 1	*TOB1*	202704_at	92.69	6.79	5.78
**p53-inducible cell-survival factor**	*P53CSV*	218403_at	48.33	6.50	7.75
Sortilin-related receptor, L(DLR class)	*SORL1*	203509_at	15.66	6.34	1.70
**Fas (TNF receptor superfamily, member 6)**	*FAS*	216252_x_at	44.31	6.23	4.54
**Ribonucleotide reductase M1 polypeptide**	*RRM1*	201477_s_at	46.58	6.19	0.41
Archaemetzincins-2	*AMZ2*	218167_at	37.48	6.16	1.22
Galactose-3-O-sulfotransferase 4	*GAL3ST4*	219815_at	38.62	5.97	3.12
**Growth arrest and DNA-damage-inducible, alpha**	*GADD45A*	203725_at	84.23	5.89	11.05
**Hypothetical protein FLJ11259**	*FLJ11259*	218627_at	7.23	5.87	3.56
Major histocompatibility complex, class I, B	*HLA-B*	209140_x_at	89.77	5.79	0.63
Testis specific, 10	*TSGA10*	220623_s_at	20.85	5.67	0.47
Hypothetical protein MDS025	*MDS025*	218288_s_at	31.35	5.66	2.38
**TP53 activated protein 1**	*TP53AP1*	209917_s_at	22.22	5.65	4.05
Leukemia inhibitory factor	*LIF*	205266_at	14.86	5.62	3.42
Interferon stimulated exonuclease gene 20 kDa-like 1	*ISG20L1*	219361_s_at	48.55	5.56	5.43
Lymphoid-restricted membrane protein	*LRMP*	35974_at	42.06	5.56	3.69
Integral membrane protein 2B	*ITM2B*	217732_s_at	20.25	5.52	-0.19
**Tumor necrosis factor receptor superfamily, member 10b**	*TNFRSF10B*	210405_x_at	46.05	5.52	1.69
**REV3-like, catalytic subunit DNA polymerase zeta**	*REV3L*	208070_s_at	65.17	5.45	6.73
**TP53 activated protein 1**	*TP53AP1*	210886_x_at	30.15	5.42	2.88
**Leucine-rich repeats and death domain containing**	*LRDD*	221640_s_at	55.27	5.31	1.54
AMP-activated protein kinase, beta 1	*PRKAB1*	201835_s_at	25.45	5.27	5.92
**Nonmetastatic cells 1 (NM23A)**	*NME1*	201577_at	83.39	5.15	3.38
Tubulin, gamma 1	*TUBG1*	201714_at	41.74	5.09	0.02
Solute carrier family 7, member 6	*SLC7A6*	203579_s_at	18.59	4.98	2.56
**RAD51 homolog**	*RAD51C*	209849_s_at	21.02	4.92	1.11

Low model scores and higher Z score constitute better model fits. The data are compared with validation scores for gene sensitivity to small interfering (si)RNAp53 (higher = better). Plain text indicates genes not previously recorded as p53 targets. Bold text indicates experimentally demonstrated p53 targets.
