# Supplementary material for: Ranked prediction of p53 targets using hidden variable dynamic modeling
Source: Genome Biol. 2006 Mar 31;7(3):R25. doi: 10.1186/gb-2006-7-3-r25 (PMC1557743; doi:10.1186/gb-2006-7-3-r25)
Supplement: Additional data file 1 — A Word document giving details of the rescaling of array data, derivation of the coefficients of the differential operator, extension of model fitting to replicate measurements, and estimation of the measurement error [file gb-2006-7-3-r25-S1.doc]

**Correction of scaling mismatches.**

After MAS5.0 normalization, we noticed that some arrays were still hampered by a scaling mismatch. To correct for this we devised a rescaling algorithm based upon finding a set of constant genes (manuscript submitted). MAS5.0 summarised data was then re-normalised by multiplying the MAS5.0 value by the following rescaling factors for each microarray:

|  | 0 hours | 2 hours | 4 hours | 6 hours | 8 hours | 10 hours | 12 hours |
| --- | --- | --- | --- | --- | --- | --- | --- |
| Repl. 1 | 1.00 | 0.98 | 1.04 | 1.04 | 1.00 | 1.08 | 1.03 |
| Repl. 2 | 0.99 | 1.02 | 1.01 | 1.09 | 1.22 | 1.06 | 1.03 |
| Repl. 3 | 0.97 | 1.00 | 0.98 | 1.02 | 1.16 | 1.16 | 1.06 |

**Derivation of the coefficients of the differential operator**

In this section we give the detailed derivation of the coefficients A_ik_ of the linear function Δ_i_(**x**) which was introduced in Eq. Error: Reference source not found. These are obtained by differentiating P(t) defined in Eqs Error: Reference source not found and Error: Reference source not found.

$\frac{dP(t)}{dt}$ = E(k, p, r, t) x_k_

where

E(k, p, r, t) = $\frac{dC(k,p,r,t)}{dt}$ . (5i)

Comparing to Eq. Error: Reference source not found, we have

A_ik_ = $\left\{\begin{matrix} E(k,p,r,t_{i}) \text{for} p\leq k\leq r \\ 0 \text{otherwise} \end{matrix}\right.$

Note that typically, the bounds *p* and *r* will vary depending on the choice of the time point *t_i_*. It remains to give explicit formulae for E(k, p, r, t_i_ ). Using Eqs. (4) and (5i) we have

E(k, p, r, t_i_ ) = for k ≠ i . (5ii)

On the other hand, if t ≠ t_j_ for all j ≠ k, then

E(k, p, r, t) = ,

Evaluating at k = i and t = t_i_ gives

E(i, p, r, t_i_ ) = . (5iii)

A further refinement is to incorporate information about the slope Δ _0_ of x(t) at t_0_ . We assume that at t_0_ the levels of the transcription factors, and the genes that it regulates are at equilibrium. Furthermore, we assume that the perturbation to f (t) is at least continuous at t_0_ . Thus, we have Δ _0_ = 0. This can be incorporated into Lagrange’s formula.

We do this by introducing an extra (dummy) point (t_–1_ , x_–1_ ). Both t_–1_ and x_–1_ are arbitrary and we shall not need to determine their values explicitly. We will require that t_–1_ ≠ t_i_ for all i = p, …, r. The role of this extra point is to encode the information that Δ _0_ = 0 and all reference to (t_–1_ , x_–1_ ) will eventually cancel out. It only makes sense to use Δ _0_ = 0 in this way if the point (t_0_ , x_0_ ) is already part of the interpolation, that is if p = 0*.* We shall denote the coefficients of the approximation of the first derivative using the r + 2 points (t_0_ , x_0_ ), …, (t_r_ , x_r_ ) and (t_–1_ , x_–1_ ) by (k, 0, r, t). These are straightforward extensions of Eqs. (5ii) and (5iii):

(k, 0, r, t_i_ ) = for k ≠ i . (5iv)

(i, 0, r, t_i_ ) = . (5v)

The approximation of the first derivative at t_0_ is given by

= (k, 0, r, t_0_) x_k_ + (-1, 0, r, t_0_) x_-1_ .

By definition this is Δ_0_ = 0, so that

x_–1_ = – x_k_ .

On the other hand the approximation Δ_i_ to the first derivative at t_i_ is

= (k, 0, r, t_i_ ) x_k_ + (-1, 0, r, t_i_ ) x_-1_
 = (k, 0, r, t_i_ ) x_k_ – (-1, 0, r, t_i_ ) x_k_
 = E_0_(k, 0, r, t_i_ ) x_k_ ,

where

E_0_(k, 0, r, t_i_ ) = (k, 0, r, t_i_ ) – (-1, 0, r, t_i_ )$\frac{\tilde{E}(k,0,r,t_{0})}{\tilde{E}(-1,0,r,t_{0})}$ . (5vi)

Note that if i = 0, we obtain

E_0_(k, 0, r, t_0_ ) = 0

for all k such that 0 ≤ k ≤ r, and the remaining E_0_(k, 0, r, t_0_ ) are 0 by definition. Hence the first row of the matrix A is identically 0, which of course it has to be for consistency with our assumption that Δ_0_ = 0. In the more general case, one can simplify Eq. (5vi) using Eqs. (5iv) and (5v) to give explicit formulae for the coefficients E_0_(k, 0, r, t_i_ ) for i ≠ 0:

E_0_(k, 0, r, t_i_ ) = $\frac{1}{t_{k}-t_{i}}$ $\frac{t_{i}-t_{j}}{t_{k}-t_{j}}$ $\frac{t_{0}-t_{i}}{t_{0}-t_{k}}$
 = E(k, 0, r, t_i_ )$\frac{t_{0}-t_{i}}{t_{0}-t_{k}}$ for k ≠ 0, i

E_0_(i, 0, r, t_i_ ) = +
 = E(i, 0, r, t_i_ ) +

E_0_(0, 0, r, t_i_ ) =
 = E(0, 0, r, t_i_ )

Given the general derivation above, we have a wide choice in the precise selection of the coefficients of the matrix A in Eq. Error: Reference source not found. We could do a 3-point estimation throughout, as outlined in the previous section. At the other extreme, we could include the whole time course, i.e. perform an n-point interpolation. Using trial and error with artificially generated data, we chose to perform a symmetric 5-point interpolation (so that p = i – 2 and r = i + 2) wherever this was possible (i.e. for 2 ≤ i ≤ n–2). For i = 0, 1, n–1 and n we used the closest possible choice to this. More precisely, the choices of p and r were thus

| **i** | **p** | **r** |
| --- | --- | --- |
| 0 | 0 | 2 |
| 1 | 0 | 3 |
| … |  |  |
| 2 ≤ i ≤ n–2 | i – 2 | i + 2 |
| … |  |  |
| n–1 | N–3 | N |
| n | N–2 | N |

In our case n = 6, and we used the modified coefficients E_0_(k, 0, r, t_i_ ) for the first three rows. The matrix A was thus

Our data was sampled at t_0_ = 0, t_1_ = 2, t_2_ = 4, t_3_ = 6, t_4_ = 8, t_5_ = 10 and t_6_ = 12. Evaluating the above coefficients explicitly gives

A = .

Note that each row sums to 0.

**Extension of model fitting to replicate measurements**

As is common, the microarray experiments were carried out in triplicate. Initially, we fitted the parameters for each experiment separately. We found, however, that we obtained more robust results if all three experiments were fitted simultaneously. Specifically, we assumed, that for each gene j, the kinetic parameters B_j_, S_j_ and D_j_ were the same in each replicate experiment, but that the transcription factor profiles were different in each replicate. In the case of three replicates the parameter vector in the first phase thus becomes

**** = ( B_1_, …, B_m_, S_1_, …, S_m_, D_1_, …, D_m_, f_0,1_, …, f_n,1_, f_0,2_, …, f_n,2_, f_0,3_, …, f_n,3_ )

where f_i,a_ denotes the value of f_i_ for replicate a. In the second phase ****_j_ remains unchanged. The error function in Eq. (7) for each gene j becomes the sum of the errors for the corresponding replicates

M_j_ = + +

Here x_j,a_ is computed from the model using f_0,1_, …, f_n,1_. The definition of the total error in Eq. (8) remains unchanged. The assumption of equality across replicates introduces additional constraints and hence causes a deterioration in the overall model score both in the training and screening schemes. This is, however, counterbalanced by tighter confidence intervals for the kinetic parameters. It also explains why we were rather lenient in the model score criteria (< 100 for 18 degrees of freedom) in the screening scheme to select for candidate p53 targets and probably also why the robustness of the estimation for the sensitivity S_j_ appears to be a better predictor for p53-dependency

**Estimation of the measurement error**

To compute the merit function in Eq. (7) we require an estimate of the measurement error σ_j_(t_i_ ) for a given observed signal _j_(t_i_ ). Initially, we assumed the relative measurement error to be constant i.e. σ_j_(t_i_ ) =  _j_(t_i_ ), with typically  = 0.2 *.* However, this did not produce satisfactory results, because signals with a high amplitude are more precise than weaker signals. As a result the latter were relatively speaking over-fitted. Furthermore, this simple scheme did not take into account the fact that some microarrays (those with a high background and/or a high scaling factor) are more noisy than others. This resulted in higher model scores for those replicate time courses which had a larger number of such microarrays.

We therefore decided to estimate measurement error from the data, allowing this to vary with both the signal intensity and the microarray. In others words, we assumed that the measurement error _j,a_ for gene j in microarray a is a function G_a_ of the signal intensity _j,a_, *i.e.*

j,a = G_a_(_j,a_ ) .

Note that this dependency of measurement errors on the signal intensity has already been proposed elsewhere in various guises [43-46]. In our in-house implementation we did not attempt to represent G_a_ in terms of mathematical functions, but instead used a look-up table. Its entries were computed as follows.

Our experiment consists of *biological* triplicates: that is each microarray time course was used to measure expression time evolution for a different biological sample. It is impossible to estimate the measurement error from this. We thus resorted to publicly available data (www.affymetrix.com/support/technical/sample_data/datasets.affx). We used Affymetrix’s spike in experiment, which was performed using the same type of microarray as that used in our experiments and in which the replicates were *technical* replicates (i.e repeated measurements from the same biological sample). We denote the measurement of gene j in replicate a for this data by _j,a_ . From this spike in experiment, we select the first three microarrays (so that a = 1,2,3). For each gene j we then compute the following quantities:

j = $\frac{1}{3}$_j,a_ ,

_j_^2^ = $\frac{1}{3-1}$(_j,a_ – _j_ )^2^ .

We then divide the genes among thirty classes H_b_ (b =1, …, 30) of equal amplitude where l_b_ and u_b_ are the lower and upper bounds of these classes. We have

u_b_ = l_b+1_ b = 1, …, 29

u_b_ – *l_b_* = (log (_j_ ) – log (_j_ )) *b* = 1, …, 30

and therefore

*u*_30_ = log (_j_ ) ,

*l*_1_ = log (_j_ ) .

We then say that a gene *j* belongs to a class H_b_ if l_b_ ≤ log (_j_ ) ≤ u_b_ and call m_b_ the total number of genes belonging to the class H_b_ . We finally define thirty pairs of values x_b_ and σ_b_ as follows, for b = 1, …, 30:

log(x_b_) = $\frac{1}{2}$(u_b_ – *l_b_*) ,

σ_b_^2^ = $\frac{1}{m_{b}}\sum_{j\in H_{b}}$_j_^2^

We thus have a measurement error estimate for thirty centres of classes which we are going to use to calibrate our own data.

With our own data, we have ñ microarrays in total (a = 1,…, ñ), including different time-points and possibly replicates. We determine the following quantities for each gene j:

j = $\frac{1}{\tilde{n}}\sum_{a}^{\tilde{n}} \hat{x}_{j,a}$ ,

_j,a_^2^ = (_j,a_ – _j_ )^2^ a = 1,…, ñ

We proceed by using the same classes defined above and say that a gene *j* belongs to a class H_b_ if l_b_ ≤ log (_j_ ) ≤ u_b_ and call _b_ the total number of genes belonging to the class H_b_ . We then define for each class H_b_ and microarray a:

_b,a_^2^ = _j,a_^2^

We thus have for each microarray a first rough estimate _b,a_ for the measurement error for each centre of the class which we need to connect with the more accurate estimate we made with the spike-in experiment. We select among our own experiment a subset of *ñ'* microarrays with a low background and low scaling factor (in practice, we selected 10 out of 21 microarrays). This subset, denoted by *J*, is therefore composed of less noisy microarrays and we assume they have a measurement error similar to the one observed in the spike-in data. We proceed by determining, for each class H_b_:

_b_^2^ =

R_b_ = .

Finally, we obtain for each microarray a in our experiment for each of its classes H_b_

σ_b,a_^2^ = R_b_ _b,a_^2^

The quantity σ_b,a_ represents our final estimate for the measurement error in microarray *a* for a signal _j,a_ whose logarithm would have the exact value of the centre of class of class H_b_ defined above, in other words _j,a_ = σ_b,a_ if _j,a_ = x_b_ . In practice, most of the signals we observe do not coincide with the centre of classes, so that we need a last step to compute the measurement error for an arbitrary signal _j,a_ . It is obtained by linear interpolation between the two centres of class adjacent to log(_j,a_), which we denote by log(x_b_*'* ) and log(x_b_*'*_+1_ ). We have:

j,a =

where

y_b_*'* = log (x_b_*'* ) ,

y_b_*_'+_*_1_ = log (x_b_*'*_+1_ ) ,

j,a = log(_j,a_).
